# Multivisceral Resection with Performing a Double Roux-en-Y Reconstruction for Advanced Gastric Cancer

**DOI:** 10.1155/2015/649723

**Published:** 2015-11-24

**Authors:** Zijah Rifatbegovic, Zlatan Mehmedovic, Majda Mehmedovic, Jasmin Hasanovic, Amra Mestric

**Affiliations:** ^1^Department of General Abdominal Surgery, Clinic for Surgery, University Clinical Center Tuzla, Tuzla, Bosnia and Herzegovina; ^2^Department of Gastroenterology and Hepatology, Clinic for Internal Diseases, University Clinical Center Tuzla, Tuzla, Bosnia and Herzegovina

## Abstract

*Background*. The role of multivisceral resection, in the setting of locally advanced gastric cancer, is still debated. Previous studies have reported a higher risk for perioperative morbidity and mortality, with limited objective benefit in terms of survival.* Patient.* A male patient, 55 years old, was admitted to the clinic of surgery for surgical treatment of bleeding gastric ulceration. Preoperative diagnostic evaluation was performed, and patient had undergone a surgical treatment which revealed a large mass in head of the pancreas, infiltrating the hepatoduodenal ligament and transverse mesocolon. Total gastrectomy, duodenopancreatectomy, and right hemicolectomy were performed. The digestive tube continuity was reestablished by deriving the double Roux limbs.* Conclusion*. The aim of this case presentation is to demonstrate a method of digestive tube reconstruction by performing the double Roux-en-Y reconstruction in advanced gastric cancer when the multivisceral resection is performed.

## 1. Introduction

Although the incidence of gastric carcinoma is declining, it is still one of the leading causes of death from malignant tumors worldwide. Despite improved diagnostic techniques, most patients present with an advanced stage tumor [1].

The role of multivisceral resection, in the setting of locally advanced gastric cancer, is still debated. Previous studies have reported a higher risk for perioperative morbidity and mortality, with limited objective benefit in terms of survival. Conversely, recent studies have shown the feasibility of enlarged resection for clinical stage T4b gastric adenocarcinoma with good long-term results [2].

For patients undergoing curative surgery for gastric cancer R0 resection (negative microscopic and macroscopic margins) is the most powerful predictor of outcome. Unfortunately, gastric cancer typically presents at an advanced stage, and tumor invasion into adjacent structures is present in many of these patients [3].

After the multivisceral resection is performed the digestive tube continuity can be made by the Roux-en-Y reconstruction, or the double Roux-en-Y, as an alternative reconstruction after the pancreaticoduodenectomy. When the pancreaticojejunostomy and hepaticojejunostomy due to pancreaticoduodenectomy are performed, the double Roux-en-Y digestive tract reconstruction can be addressed to decrease the liquid flow and pressure in the duodenal lumen and reduce not only the distance between the pancreaticojejunostomy and choledochojejunostomy but also the risks of traction, twisting, and angularity of the jejunal loop associated with common reconstruction methods [4].

We aim to present a case of 56-year-old male who underwent surgery for an advanced gastric cancer, as well as the optimal method of digestive tube reconstruction following a multivisceral resection.

## 2. Case Presentation

**Figure 1:**
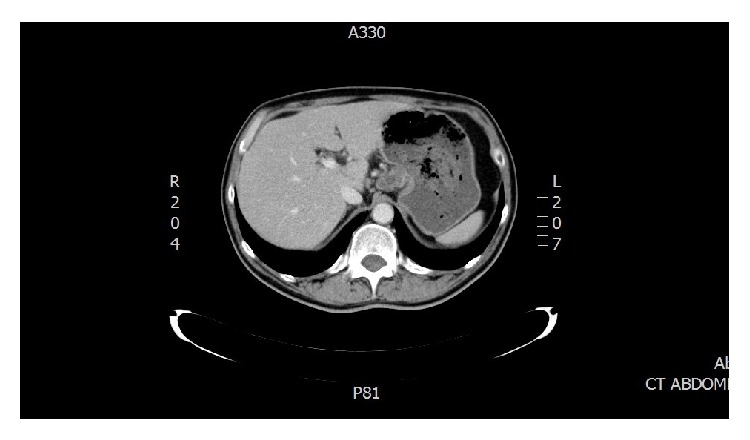
Computed tomography scan (CT) showed a structure of 2.0 × 3.5 cm in diameter on a lesser gastric curvature that could stand for large penetrating ulcer.

A male patient, 55 years of age, was admitted to surgery clinic for surgical treatment of bleeding gastric ulcer. His major complaints were fatigue, abdominal pain, and tarry stool. Physical examination revealed painful abdomen in the region of epigastrium. During esophagogastroduodenoscopy gastric lumen was filled with multiple coagulum and revealed an elongated deep ulcer that stretched out from lesser gastric curvature to middle and lower third of gastric body. Its borders reddened and its bottom was filled with fibrin and coagula. Computed tomography scan (CT) showed a structure of 2.0 × 3.5 cm in diameter on a lesser gastric curvature that could stand for large penetrating ulcer (Figure 1).

After preoperative diagnostics was performed, patient underwent a surgical treatment and a large tumour that infiltrates head of pancreas, hepatoduodenal ligament, and transverse mesocolon was found. Total gastrectomy, duodenopancreatectomy, and right hemicolectomy were performed. The procedure took five hours and thirty minutes.

The pathohistological analysis showed margins which are tumor-free and confirmed that the R0 resection was performed. Due to pathological T4 (TNM stage) of the tumor omentectomy and D1 lymphadenectomy were also performed.

**Figure 2:**
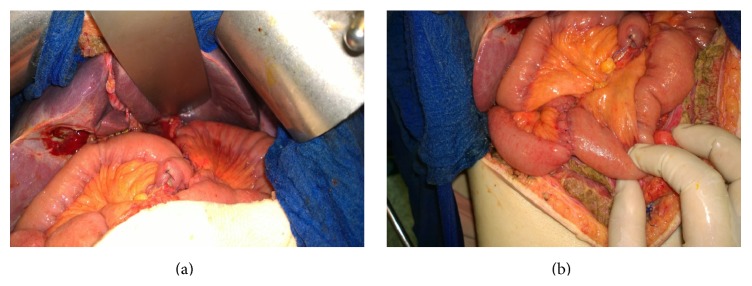
(a) and (b) show esophagojejunal anastomosis, hepaticojejunal, pancreaticojejunal, and jejunojejunal anastomoses.

The continuity of digestive tube was reestablished by deriving double Roux limbs. Jejunal limb was isolated by Roux method and end-to-side esophagojejunal anastomosis by circular stapler was made. The limb was then resected on 60 cm from esophagojejunal anastomosis. Digestive tube continuity was reestablished by creating hepaticojejunal, pancreaticojejunal, and jejunojejunal anastomoses. Ileum-transversum anastomosis was created as well (Figures 2(a) and 2(b)).

Complications such as bile leakage, pancreatic leakage, and digestive tract obstruction were not observed during the follow-up period.

## 3. Discussion

The overall 5-year survival rate for all patients with gastric carcinoma who underwent surgery is only 20–30%. Even after a curative resection, only 30–50% of the patients are still alive after 5 years, with local recurrence being the main cause of treatment failure. The surgical management of T4 gastric carcinoma remains controversial and the benefits of an extended resection are still doubtful [5].

The prognosis of patients with gastric cancer with invasion to adjacent organs (T4) was reported to be poor. However, some patients treated by radical surgery can survive for a long time after surgery [6].

For gastric carcinoma, standard therapy included subtotal/total gastrectomy, D2 lymphadenectomy, and omentectomy. The extent of the resection performed depended on tumor site and histological type. If safe resection margins could be achieved, subtotal gastrectomy was performed. If pancreatic infiltration was suspected intraoperatively, en bloc gastrectomy with pancreatic resection was performed. Splenectomy was done in patients with proximal carcinoma invading the spleen, in cases of enlarged lymph nodes in the splenic hilum, or in cases with invasion of the pancreas tail or body (in combination with pancreatic resection) [1].

Method for digestive tube reconstruction after multivisceral resection remains an open question. There are different ways to create reconstruction. In our case, we decided for a double Roux limb to avoid performing all the necessary anastomoses on a single loop.

When compared to single Roux limb, double Roux-en-Y reconstruction of the digestive tube is not beneficial in terms of surgical outcome and postoperative morbidity and mortality and should be avoided due to unnecessarily prolonged surgery [7].

We believe that in certain cases performing the double Roux-en-Y reconstruction can be a successful way to address the digestive tube continuity.

## 4. Conclusion

Even though the previous studies about the double Roux-en-Y showed no beneficial surgical outcome, in our case report it was a successful way for digestive tube reconstruction. We believe that the double Roux-en-Y should be considered as a way for reconstruction in certain cases with a multivisceral resection.
